# THE IMPACT OF SOCIAL DEPRIVATION ON COVID-19 INFECTIONS IN NURSING HOMES

**DOI:** 10.1093/geroni/igac059.1563

**Published:** 2022-12-20

**Authors:** Robert Weech-Maldonado, Justin Lord, Ganisher Davlyatov, Akbar Ghiasi, Gregory Orewa

**Affiliations:** University of Alabama at Birmingham, Birmingham, Alabama, United States; Louisiana State University at Shreveport, Bossier City, Louisiana, United States; University of Oklahoma, Oklahoma City, Oklahoma, United States; University of the Incarnate Word, San Antonio, Texas, United States; University of Alabama at Birmingham, Birmingham, Alabama, United States

## Abstract

Health inequities vary along social and economic gradients. The COVID-19 pandemic and nursing home infections have highlighted this fact. Using the Centers for Medicare and Medicaid Services’ Nursing Home COVID-19 Public File, Brown University’s LTCFocus, Robert Graham Center’s Social Deprivation Index, and CMS Nursing Home Payroll Based Journal Staffing Data. We examined the relationship between community resource scarcity, as conceptualized by the Social Deprivation Index (SD) and COVID-19 incidence rates in nursing homes. After controlling for interstate differences, organizational enabling factors, as well as, facility-level resident and community-level characteristics, nursing homes located in communities with medium levels of social deprivation had 4.4% more COVID-19 infection rates (Incidence Rate Ratio [IRR] = 1.04; p < 0.05) and communities with high levels of social deprivation had 7.5% higher COVID-19 infection rates (Incidence Rate Ratio [IRR] = 1.07; p < 0.01) as compared to nursing facilities located in areas of low social deprivation. From a policy perspective, nursing homes, that are located in socially deprived communities, may need additional resources, such as, funding for staffing and personal protective equipment in the face of the pandemic. The COVID-19 pandemic has sharpened the focus on healthcare disparities and societal inequalities in the delivery of long-term care.

